# Ocean acidification impacts spine integrity but not regenerative capacity of spines and tube feet in adult sea urchins

**DOI:** 10.1098/rsos.170140

**Published:** 2017-05-17

**Authors:** Chloe E. Emerson, Helena C. Reinardy, Nicholas R. Bates, Andrea G. Bodnar

**Affiliations:** 1 Bermuda Institute of Ocean Sciences, 17 Biological Station, St George's GE 01, Bermuda; 2 Department of Ocean and Earth Science, National Oceanography Centre, University of Southampton, Southampton, UK

**Keywords:** sea urchin, spine, tube feet, regeneration, biomineralization, ocean acidification

## Abstract

Increasing atmospheric carbon dioxide (CO_2_) has resulted in a change in seawater chemistry and lowering of pH, referred to as ocean acidification. Understanding how different organisms and processes respond to ocean acidification is vital to predict how marine ecosystems will be altered under future scenarios of continued environmental change. Regenerative processes involving biomineralization in marine calcifiers such as sea urchins are predicted to be especially vulnerable. In this study, the effect of ocean acidification on regeneration of external appendages (spines and tube feet) was investigated in the sea urchin *Lytechinus variegatus* exposed to ambient (546 µatm), intermediate (1027 µatm) and high (1841 µatm) partial pressure of CO_2_ (*p*CO_2_) for eight weeks. The rate of regeneration was maintained in spines and tube feet throughout two periods of amputation and regrowth under conditions of elevated *p*CO_2_. Increased expression of several biomineralization-related genes indicated molecular compensatory mechanisms; however, the structural integrity of both regenerating and homeostatic spines was compromised in high *p*CO_2_ conditions. Indicators of physiological fitness (righting response, growth rate, coelomocyte concentration and composition) were not affected by increasing *p*CO_2_, but compromised spine integrity is likely to have negative consequences for defence capabilities and therefore survival of these ecologically and economically important organisms.

## 1. Introduction

The increase in atmospheric carbon dioxide (CO_2_) levels over the past century due to anthropogenic activities has led to increased uptake of CO_2_ into the ocean, and changes in CO_2_-carbonate chemistry commonly termed ocean acidification [1,2]. Dissolved CO_2_ interacts with seawater and is converted into carbonic acid (H_2_CO_3_), which then dissociates into bicarbonate (HCO_3_^−^), carbonate (CO_3_^2−^) and hydrogen (H^+^) ions, resulting in a corresponding decrease in oceanic pH and calcium carbonate (CaCO_3_) saturation state (Ω) [2]. Changing ocean chemistry as a consequence of ocean acidification has the potential to directly and indirectly impact organisms, particularly marine calcifiers that build and maintain CaCO_3_ shells and other body structures [3].

Sea urchins are ecologically and economically important marine calcifiers that may be particularly vulnerable to ocean acidification because their skeletal components are composed of high magnesium calcite (Mg-calcite), which is more soluble than low Mg-calcite or aragonite forms of CaCO_3_ [4]. The sea urchin larval skeleton and adult test, teeth, spines and tube feet contain calcite mineral structures, each containing different levels of magnesium [5]. Previous studies have mainly focused on the effects of ocean acidification on early life stages (embryos and larvae), with fewer studies on juvenile and adult sea urchins [6,7]. Results indicate that the effects of ocean acidification are species-specific, process-specific and life-stage specific, suggesting that it is imperative to study a range of processes throughout the life stages of these animals to predict the impact on wider populations. Studies have demonstrated negative impacts of increased *p*CO_2_ on sea urchin larval, juvenile and adult growth [6–11]; however, no previous studies have investigated the effects of increased *p*CO_2_ on the process of spine or tube feet regeneration.

Sea urchins can repeatedly regenerate external appendages (spines, tube feet and pedicellariae) that serve essential locomotory, defence and sensory functions [12–14]. Given these important functions, any compromised ability to rebuild damaged appendages under altered environmental conditions may lower their ability to effectively defend against predators, move, feed and sense the environment, which in turn may ultimately impact individual survival. Spine biomineralization is driven by skeletogenic cells (sclerocytes) located in the dermis that covers the surface of the sea urchin skeleton (an endoskeleton). Spine regeneration initially involves a wound-healing process where the epidermis is reconstituted around the broken spine. Calcification then takes place in a syncytium formed by the sclerocytes to build a single-crystal structure of Mg-calcite [12]. It has been predicted that regenerating spines are particularly vulnerable to ocean acidification because the transient amorphous CaCO_3_ precursor formed during regeneration is more soluble than the crystalline calcite of mature spines [8,15].

Sea urchin tube feet are fleshy extensions of the water vascular system that protrude through the sea urchin test and play a role in locomotion, respiration and sensory perception. A disc at the distal end of each tube foot is used for adhesion and also receives sensory input that is transduced to the radial nerve lying just inside the test [16]. The distal disc contains calcite ossicles that may serve to focus light, thereby increasing light absorption by the photoreceptor cells [16], a process that may be compromised if ocean acidification affects the formation of the disc.

Biomineralization is well studied in sea urchin embryos and larvae and is regulated by a variety of cellular signalling pathways and proteins that facilitate the process [17,18]. These include MSP130, a cell surface glycoprotein involved in the transport of Ca^2+^ to site of skeletogenesis; SM50, a spicule matrix protein; P16, a transmembrane protein that plays a role in skeletal rod elongation; carbonic anhydrase (e.g. CAHB, CARA7LA), an enzyme involved in the conversion of CO_2_ to HCO_3_^−^; and a variety of carbohydrate-binding lectins (e.g. C-lectin, C-lectin/PMC1) [17,18]. Although the cellular pathways of biomineralization in post-metamorphic sea urchins are not well characterized, the expression of similar genes and proteins has been reported in adult skeletal components, suggesting a role in later life stages [18,19].

It has been suggested that the regenerative abilities of echinoderms are one of the most important factors responsible for the evolutionary success of this phylum throughout the marine ecosystem [13] and, therefore, it is important to understand the impact of CO_2_-driven ocean acidification on these processes.

## 2. Material and methods

### 2.1. Collection and maintenance of sea urchins

*Lytechinus variegatus* were collected from Mangrove Bay in Bermuda (32°22.25′ N and 64°41.5′ W). Eighteen sea urchins (46–64 mm test diameter) were collected in July 2015 and four additional sea urchins of similar size were collected in August to serve as controls for the second amputation experiment. After collection, sea urchins were kept in flow-through aquaria (70 l) divided into six separate compartments, each containing one individual, with each experimental condition in a different aquarium (ambient, intermediate and high *p*CO_2_). The sea urchins were distributed into different aquaria such that the average test size was similar for each treatment group. Sea urchins were fed daily with a formulated sea urchin feed [20] and tanks were cleaned daily. The experimental set-up was designed to minimize tank effects by housing the animals in separate chambers that prevented direct interaction, maintaining the seawater flow rate such that the volume of each aquarium was refreshed approximately every 1.4–2 h, and monitoring seawater parameters as described below.

### 2.2. Maintenance of aquaria with different *p*CO_2_ conditions

A continuous flow-through seawater system was established with a settlement header tank (greater than 300 l) that fed three 50 l carboys, bubbled with air stones for *p*CO_2_ treatments, and siphoned into the respective aquaria at a flow rate of approximately 50 l h^−1^. Exposure conditions were established by mixing air (ambient condition) and CO_2_ (intermediate and high *p*CO_2_ treatments) by GFC mass flow controllers (Aalborg, New York, USA). Water conditions were monitored three to six times daily for pH, conductivity, temperature and salinity with a YSI Pro1030 (YSI Inc., Yellow Springs, OH, USA). Ammonia (NH_3_/NH_4_^+^) and nitrate (NO_3_^−^) levels were monitored weekly according to the manufacturer's instructions using API test kits (API Aquarium Pharmaceuticals/MARS Fishcare, Chalfont, PA, USA), and dissolved oxygen was measured weekly with a DO200A instrument (YSI Inc.). Water was sampled weekly for chemical analysis of dissolved inorganic carbon (DIC) and total alkalinity (TA) (Versatile INstrument for the Determination of Titration Alkalinity, VINDTA-3C system, Marianda, Kiel, Germany) following standard protocols [21]. The precision of DIC and TA was greater than 1 µmol kg^−1^ (approx. 0.05%) and accuracy of approximately 1 µmol kg^−1^ was maintained with routine analysis of seawater certified reference materials. pH, *p*CO_2_, [CO_3_^2−^] and saturation states (Ω) for aragonite, calcite and high magnesium calcite were calculated using CO2SYS software [22], using carbonic acid and boric acid dissociation constants [23--25].

### 2.3. Sea urchin tissue regeneration assay

**Figure 1.**
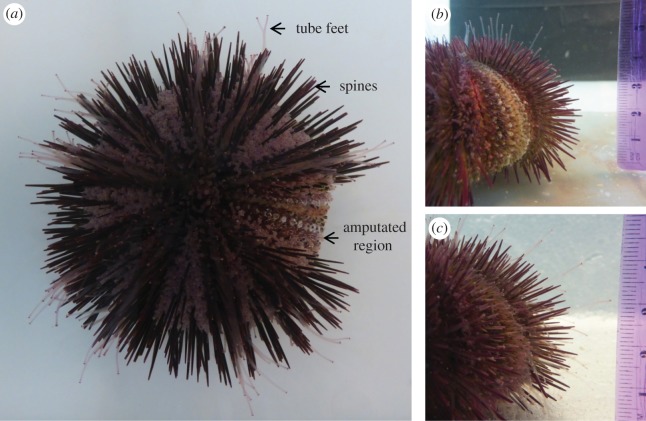
Sea urchin tube feet and spine regeneration assay. (*a*) Aboral view 1 day post-amputation showing tube feet and spines removed from along one of the ambulacral segments of the test from the oral to aboral surface. (*b*) Lateral view of the amputated region, 1 day post-amputation. (*c*) Lateral view of the amputated region, 8 days post-amputation.

Tube feet and spine regeneration were measured by the sea urchin regeneration assay as described by Reinardy *et al.* [14] and illustrated in figure 1. Briefly, a single strip of tube feet and surrounding spines were amputated from one ambulacral section. Spines and tube feet were measured weekly (8, 15, 22 and 29 days post-amputation) and subsequently re-amputated and measured each week for a further four weeks corresponding to 38, 45, 52 and 59 days in total. An additional ambient control treatment tank with newly collected and amputated sea urchins (*n* = 4) was introduced at the start of the second period of regeneration. Ten regenerating spines along the amputated area and 10 full-length spines in the adjacent, uncut area were measured weekly using callipers and spine regeneration was calculated at each time point using the mean regenerating spine length relative to mean full length spines. Tube feet were measured by image analysis (‘Fiji is Just ImageJ’ software, http://fiji.sc/Fiji [26]) of underwater photographs taken weekly, and regeneration was calculated using regenerating tube feet measurements (*n* = 10) relative to full-length tube feet (*n* = 10) measured in the same photograph. Percentage regeneration data were arcsine transformed, and the overall effect of time and *p*CO_2_ treatment on regeneration was tested by general linear model (GLM); within time point concentration differences were tested by one-way ANOVA with *post hoc* multiple range tests.

### 2.4. Structural analysis and elemental composition of spines

Regenerating spines were collected from the amputated strip (at 59 days exposure) and homeostatic spines were collected from an adjacent ambulacral section. Prior to analysis, spines were immersed in 4% sodium hypochlorite for 1 h, rinsed three times with water and dried. Structure of the homeostatic and regenerating spines collected from three sea urchins in the ambient and three sea urchins in the high *p*CO_2_ treatment groups was investigated using a Zeiss SUPRA 40vp Gemini Scanning Electron Microscope in the Central Microscopy Facility at the Marine Biological Laboratory (MBL) in Woods Hole, MA, USA. Spines were attached to aluminium specimen mount stubs (Ted Pella, Inc., Redding, CA, USA) and sputter coated with 6 mm layer of platinum using a Leica MED 020 high vacuum coating system. Images were captured with the Zeiss SMARTSEM software. Elemental composition of the spines (*n* = 3 animals from each of the ambient and high *p*CO_2_ conditions, technical replicates of three spines per individual) was determined using inductively coupled plasma mass spectrometry (ICP-MS) on the Element 2 ICP-MS (Thermo Scientific, Waltham, MA, USA) in the Plasma Mass Spectrometry Facility at Woods Hole Oceanographic Institution (WHOI). All spines were trimmed to 1 cm length from the tip, weighed and dissolved in 4 ml of 5% HNO_3_ and 1 ppb In was added to all samples and standards as an internal control. Three standard certified reference material samples were used for the standard curve calculation: aragonite fish otolith (FEBS-1, National Research Council, Canada), carbonate standard (MACS-3, United States Geological Survey, USA) and aragonite coral (JCp-1, [27]). Ratios of Mg, Ba, Li, and Sr with Ca were calculated from the reference standards calibration curve.

### 2.5. Spine load-bearing test

Spine fragility was measured with a mechanical loading assay called the ‘spine snap test’ using a homemade device shown in electronic supplementary material, figure S1. Bleached and dried spines were trimmed to a length of 9 mm measured from the tip and balanced between two platforms so that 1 mm rested on each platform and 7 mm was left unsupported (electronic supplementary material, figure S1). A receptacle was balanced directly in the middle of the spine (4.5 mm from the tip) and weight was added incrementally to this receptacle until the spine broke. The receptacle and load were weighed to determine the total weight required to break the spine (load-bearing weight). Replicates (*n* = 3–10 spines) were averaged for each animal and the data were analysed using a one-way ANOVA to compare mean values between homeostatic and regenerating spines within each treatment group. The mean values were also compared between treatment groups (ambient, intermediate and high *p*CO_2_) for homeostatic or regenerating spines.

### 2.6. Gene expression analysis

At 29 and 59 days exposure to the different *p*CO_2_ conditions, regenerating spines and tube feet were collected into RNA*later* solution (Qiagen, Valencia, CA, USA) and stored at −80°C. RNA was extracted from the tube feet and spines using Trizol reagent (Invitrogen, Carlsbad, CA, USA) followed by the RNA clean-up protocol of the RNeasy mini kit (Qiagen). cDNA was synthesized (High-Capacity cDNA Reverse Transcription Kit, Applied Biosystems, Foster City, CA, USA) and analysed by quantitative reverse transcription–PCR using the SYBR Green detection system on the ABI 7300 Real Time-PCR machine (Applied Biosystems). Primers were designed using Primer Express software (v. 3.0, Applied Biosystems) with sequences of *L. variegatus* genes identified in the echinoderm genome database (www.echinobase.org) (electronic supplementary material, table S1). Differential expression of target genes (*msp130*, *c-lectin*, *c-lectin/pmc1*, *sm50*, *cahb, cara7la*, *P16*) was determined using the ΔΔCt method normalized to three stable control genes (*profilin, rpl8 and cyclophilin-7*) [28]. Non-significant treatment effects on control gene expression were verified by one-way ANOVA (*p* > 0.05). Treatment effects on relative fold change in gene expression were tested by one-way ANOVA where the data complied with normality and the Kruskal–Wallis test for non-parametric data.

### 2.7. Physiological state

For general indication of physiological state, righting response (time taken to ‘right’ to a normal position following inversion) was measured after 29 and 58 days exposure (three trials per individual at both time points). Weight of each sea urchin was measured prior to amputation at 0 days exposure and after 58 days exposure. Coelomic fluid (200 µl) was collected using a 21 G needle through the peristomial membrane at 29 and 58 days exposure, respectively. Total cell concentration and differential cell counts (red cells relative to other coelomocytes) were evaluated using a haemocytometer (Neubauer Bright Line). The mean values were compared between treatments using a one-way ANOVA with *post hoc* analysis.

## 3. Results

### 3.1. Seawater CO_2_-carbonate chemistry

Seawater CO_2_-carbonate chemistry parameters for each of the three experimental conditions (ambient, intermediate and high *p*CO_2_), averaged over the entire experiment, are summarized in table 1. Temperature, salinity, conductivity and pH were measured three to six times daily, while DIC and TA were measured weekly in order to calculate pH, *p*CO_2_ and saturation states of calcite, aragonite and Mg-calcite. The mean *p*CO_2_ and pH were significantly different between the ambient, intermediate and high treatment groups (one-way ANOVA, *p* < 0.05). Calcite, aragonite and Mg-calcite saturation states (Ω) decreased with increasing *p*CO_2_, with Mg-calcite Ω [29] estimated at 1.5 ± 0.2 and 0.85 ± 0.2 in the intermediate and high *p*CO_2_ treatment groups, respectively. Multiple daily measurements of pH show some fluctuations throughout the eight-week exposure, but throughout the experiment, a clear difference in pH was maintained between treatment groups. This was verified by weekly analytical chemistry measurements and calculated pH values (electronic supplementary material, figure S2). Conductivity, salinity and temperature fluctuated daily to a small extent, but these fluctuations occurred consistently in all three experimental conditions (electronic supplementary material, figure S3).

**Table 1.** Average measured water parameters over 62 days in aquaria with ambient, intermediate and high *p*CO_2_ conditions. Parameters measured three to six times daily by YSI meter (*n* = 157, temperature, salinity, conductivity), weekly by analytical chemistry (*n* = 10, DIC and TA, VINDTA33C system), or calculated with CO2SYS software (pH, *p*CO_2_, saturation state of calcite (Ω_C_), aragonite (Ω_A_) and high magnesium calcite (Ω_MC_)). Data are means ± s.e.m.

treatment	temperature (°C)	salinity (ppt)	conductivity (µS cm^−1^)	DIC (µmol kg^−1^)	TA (µmol kg^−1^)	pH	*p*CO_2_ (µatm)	Ω_C_	Ω_A_	Ω_MC_
ambient	28.3 ± 0.03	37.14 ± 0.02	59 548 ± 49	2031 ± 5	2306 ± 6	7.93 ± 0.01	546 ± 9	4.83 ± 0.11	3.23 ± 0.07	2.40 ± 0.2
intermediate	28.0 ± 0.04	37.15 ± 0.02	59 330 ± 59	2143 ± 12	2302 ± 5	7.70 ± 0.03	1027 ± 69	3.15 ± 0.19	2.11 ± 0.13	1.50 ± 0.2
high	28.0 ± 0.03	37.13 ± 0.02	59 188 ± 54	2236 ± 9	2304 ± 6	7.47 ± 0.02	1841 ± 93	1.96 ± 0.12	1.31 ± 0.08	0.85 ± 0.2

### 3.2. Tissue regeneration

**Figure 2.**
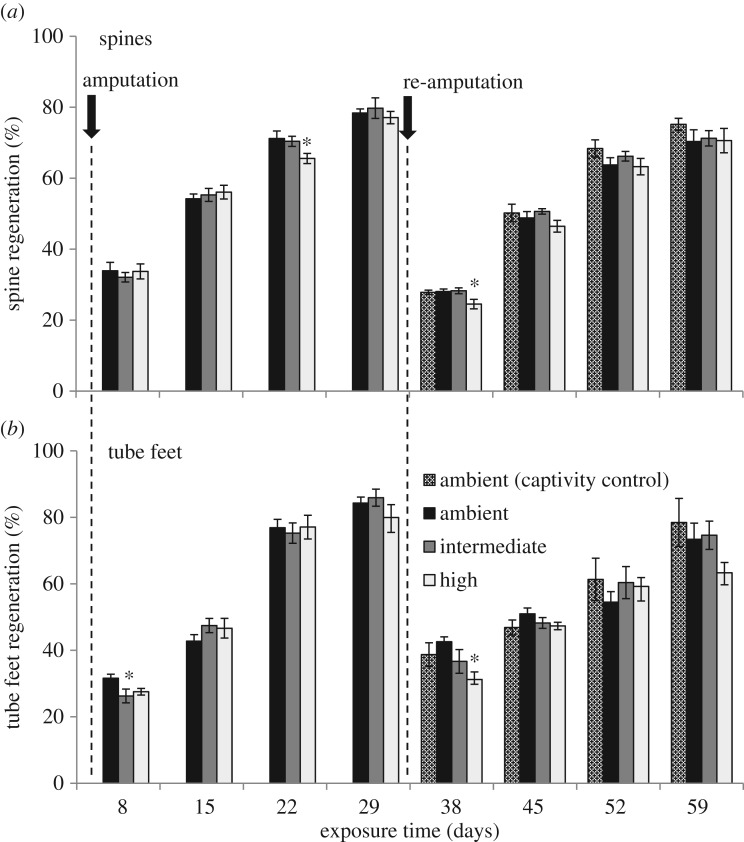
Spine and tube feet regeneration in sea urchins exposed to elevated *p*CO_2_. Regeneration (% of full length) of amputated spines (*a*) and tube feet (*b*) in adult sea urchins exposed to ambient conditions (black bars), intermediate *p*CO_2_ (dark grey bars) and high *p*CO_2_ (light grey bars) over 59 days exposure. Arrows and vertical dashed lines indicate initial amputation and re-amputation, and dotted bars indicate first amputation of ambient captivity control treatment. Data are means ± s.e.m., *n* = 6 (except *n* = 4 for the ambient captivity control and *n* = 5 for tube feet intermediate treatment group after 29 days exposure); no overall treatment effect (GLM, *p* > 0.05) and (*) significantly different from ambient treatment, *post hoc* multiple range test.

The effect of increased *p*CO_2_ on tissue regeneration in *L. variegatus* was assessed using the spine and tube feet regeneration assay described by Reinardy *et al.* [14] and illustrated in figure 1. Following amputation, the rate of spine regeneration, expressed as a percentage of full-length (not amputated) spines, was generally unaffected by elevated *p*CO_2_ (figure 2*a*, arcsine-transformed data, one-way ANOVA, *p* > 0.05). There was a small, but significant decrease in spine regeneration at days 22 and 38 in the high *p*CO_2_ treatment group compared with the ambient controls (figure 2*a*, arcsine-transformed data, one-way ANOVA, *p* < 0.05, *post hoc* Fisher's LSD), but this difference did not persist in subsequent measurements. At 29 days of exposure, all treatment groups had reached 77–80% regeneration (figure 2*a*). Following a second amputation, all groups had reached 70–75% regeneration by 59 days of exposure (figure 2*a*). Differences within treatment groups between 29- and 59-day exposure were non-significant (arcsine-transformed data, one-way ANOVA, *p* > 0.05). The overall rate of spine regeneration was 0.37 ± 0.01 mm d^−1^, similar to previously reported values for *L. variegatus* [14].

The rate of tube feet regeneration following amputation, expressed as a percentage of full-length (not amputated) tube feet, was generally unaffected by elevated *p*CO_2_ (figure 2*b*). There was a small, but significant decrease in tube feet regeneration at 8-day exposure in the intermediate treatment group and at 38-day exposure in the high *p*CO_2_ group compared with the ambient controls (figure 2*b*, arcsine-transformed data, one-way ANOVA, *p* < 0.05, *post hoc* Fisher's LSD), but these differences did not persist in subsequent measurements. At 29 days of exposure, all treatment groups reached 80–86% regeneration, and at 59 days of exposure, all groups reached 63–78% regeneration with no significant differences within treatment groups between days 29 and 59. The overall rate of tube feet regeneration was 0.72 ± 0.06 mm d^−1^, similar to previously reported values for *L. variegatus* [14].

There was no significant difference in the rate of spine or tube feet regeneration between the (ambient) captivity control and the other treatment groups in the re-amputation trial, indicating that regenerative capacity was not compromised by the repeat amputation.

### 3.3. Structural and elemental analyses of spines

**Figure 3.**
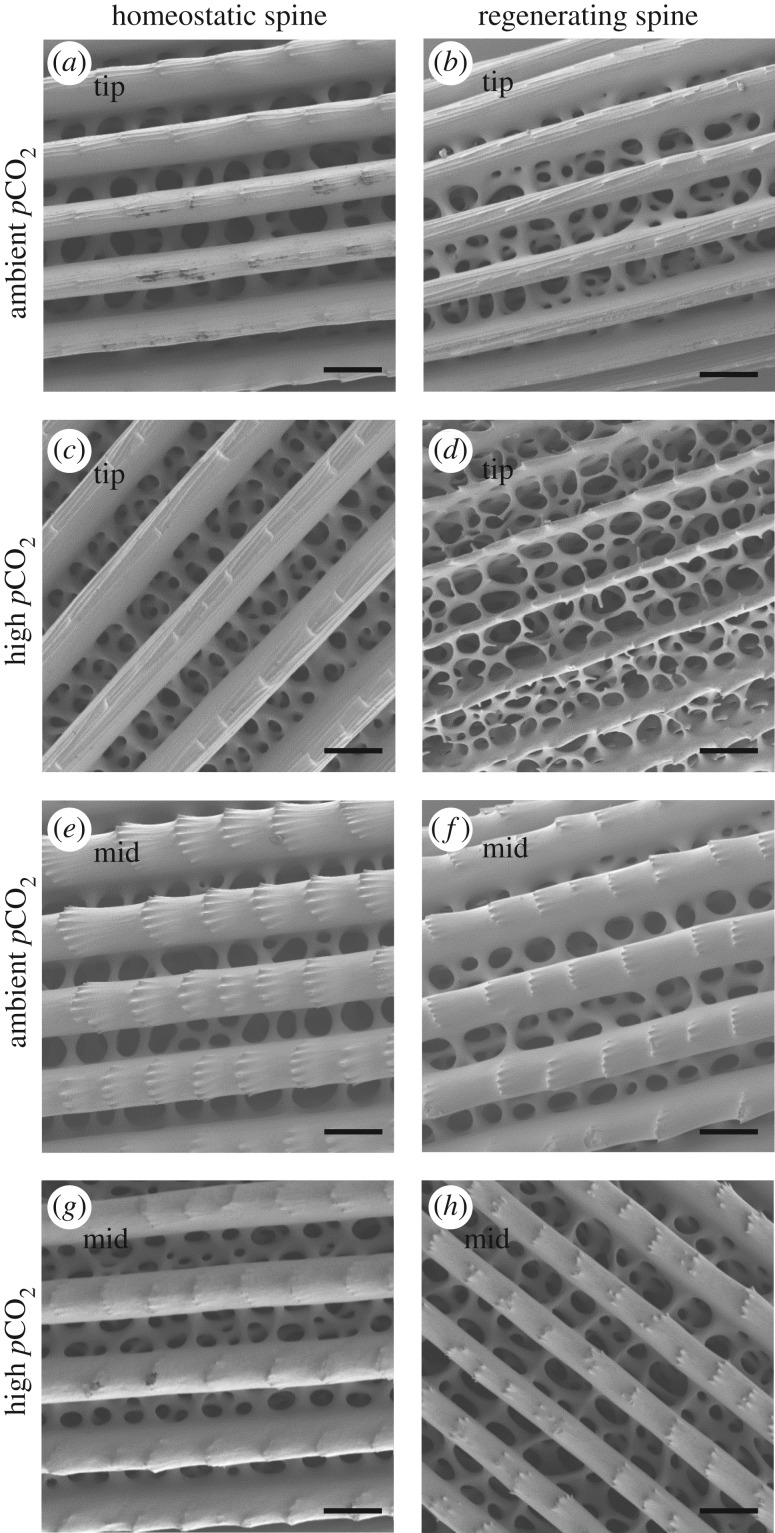
Scanning electron micrographs of spines from ambient and high *p*CO_2_ conditions. (*a*,*e*) Homeostatic spine from ambient conditions. (*b*,*f*) Regenerating spine from ambient conditions. (*c*,*g*) Homeostatic spine from high *p*CO_2_ conditions. (*d*,*h*) Regenerating spine from high *p*CO_2_ conditions. (*a*–*d*) are about 1 mm from the tip of the spine (tip). (*e*–*f*) are about 5 mm from the tip of the spine (mid). Scale bar represents 50 µm. Images were selected as representatives of homeostatic and regenerating spines from *n* = 3 sea urchins for each of the ambient and high *p*CO_2_ conditions.

Although the rate of spine regeneration was maintained under conditions of elevated *p*CO_2_, it was noted that the spines of the animals in the highest treatment group were brittle and easily damaged with normal handling. Scanning electron microscopy (SEM) was used to investigate the structural integrity of the regenerating spines (at 59 days exposure) and homeostatic spines collected from three sea urchins in the ambient and three sea urchins in the high *p*CO_2_ treatment groups. The regenerating spines had larger pores in the stereom mesh and thinner septa compared with homeostatic spines, a difference that was particularly evident in the high *p*CO_2_ conditions and most evident near the tip of the spine (figure 3). Consistent with the SEM results, the weight of the 59-day regenerating spines (mg cm^−1^) was significantly less than that of the homeostatic spines in each *p*CO_2_ condition (table 2). There was a noticeable reduction in the sharpness of the external barbs on homeostatic spines from the high *p*CO_2_ conditions compared with ambient conditions (figure 3, compare *e* and *g*). Elemental composition analysis conducted on spines that were trimmed to a standardized length (1 cm from the tip) revealed a higher magnesium to calcium ratio in regenerating spines; however, only the difference between homeostatic and regenerating spines from the high *p*CO_2_ conditions was significantly different (table 2). The content of magnesium in the trimmed spines ranged from 5.04 to 5.63 mol% (table 2). The calcium ratios of the other elements (Sr, Ba and Li) showed no significant changes with regeneration or treatment, except for regenerating spines which had lower Li/Ca, irrespective of *p*CO_2_ condition (table 2).

**Table 2.** Weight and elemental analysis of homeostatic and regenerating spines from ambient and high *p*CO_2_ conditions, determined by ICP-MS. Data are mean ± s.e.m., *n* = 3 animals per treatment condition (technical replicates of three spines per individual). Ratios are calculated from standard curves of three certified reference samples (MACS-3, FEBS-1 and JCp-1).

	homeostatic		regenerating	
	ambient	high *p*CO_2_	ambient	high *p*CO_2_
spine wt (mg cm^−1^)	^a^4.91 ± 0.33	^a^5.64 ± 0.67	^b^3.07 ± 0.28	^b^2.43 ± 0.18
Mg/Ca (mmol mol^−1^)	^ab^56.39 ± 0.70	^b^53.17 ± 1.37	^a^58.62 ± 0.82	^a^59.79 ± 1.62
Mg (mol%)	^ab^5.33 ± 0.06	^b^5.04 ± 0.12	^a^5.52 ± 0.07	^a^5.63 ± 0.14
Sr/Ca (mmol mol^−1^)	2.39 ± 0.03	2.39 ± 0.03	2.39 ± 0.02	2.37 ± 0.03
Ba/Ca (mmol mol^−1^)	^ab^2.4 × 10^−3^ ± 6.2 × 10^−5^	^b^2.6 × 10^−3^ ± 6.2 × 10^−5^	^a^2.4 × 10^−3^ ± 3.6 × 10^−5^	^ab^2.5 × 10^−3^ ± 5.7 × 10^−5^
Li/Ca (mmol mol^−1^)	^a^0.045 ± 0.0011	^ac^0.044 ± 0.0005	^bc^0.044 ± 0.0003	^b^0.040 ± 0.0010

Note: The different lower case letters in each row indicate statistical differences, *p* < 0.05, one-way ANOVA.

### 3.4. Spine load-bearing test

**Figure 4.**
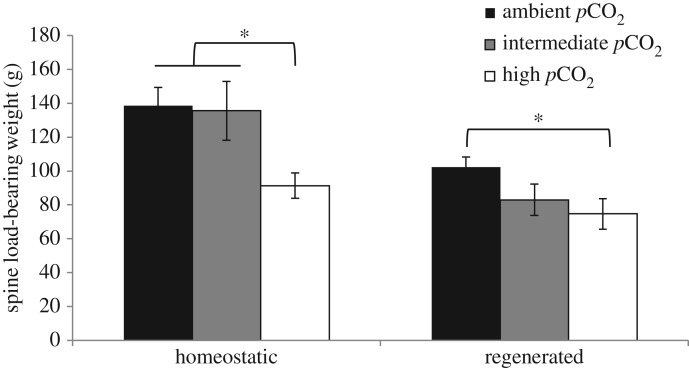
Sea urchin spine snap test after 59 days *p*CO_2_ treatment exposure period. The amount of weight required to break homeostatic and regenerating spines is shown for the three treatment groups: ambient (black bars), intermediate (grey bars) and high *p*CO_2_ (white bars). Regenerating spines were significantly weaker than homeostatic spines (GLM, *p* < 0.05), and animals from the high *p*CO_2_ treatment had significantly weaker spines (homeostatic and regenerated) compared with spines from animals kept under ambient control conditions (*one-way ANOVA, *p* < 0.05, *post hoc* multiple range test). Data are means ± s.e.m., *n* = 6 animals per treatment (technical replicates of 3–10 spines per individual).

In order to quantify the impact of elevated *p*CO_2_ on spine fragility, a mechanical loading assay (‘spine snap test’) was devised (electronic supplementary material, figure S1). The weight required to break homeostatic spines was significantly greater than the weight required to break regenerating spines in the ambient and intermediate *p*CO_2_ treatment groups (figure 4, one-way ANOVA, *p* < 0.05), but there was no significant difference in the weight required to break homeostatic and regenerating spines within the high *p*CO_2_ treatment group. Significantly less weight was required to break regenerating spines in the high *p*CO_2_ treatment group than regenerating spines in the ambient *p*CO_2_ treatment group (figure 4, one-way ANOVA, *p* < 0.05). Significantly less weight was required to break homeostatic spines in the high *p*CO_2_ treatment group than in both the ambient and intermediate *p*CO_2_ treatment groups (figure 4, one-way ANOVA, *p* < 0.05).

### 3.5. Gene expression

**Figure 5.**
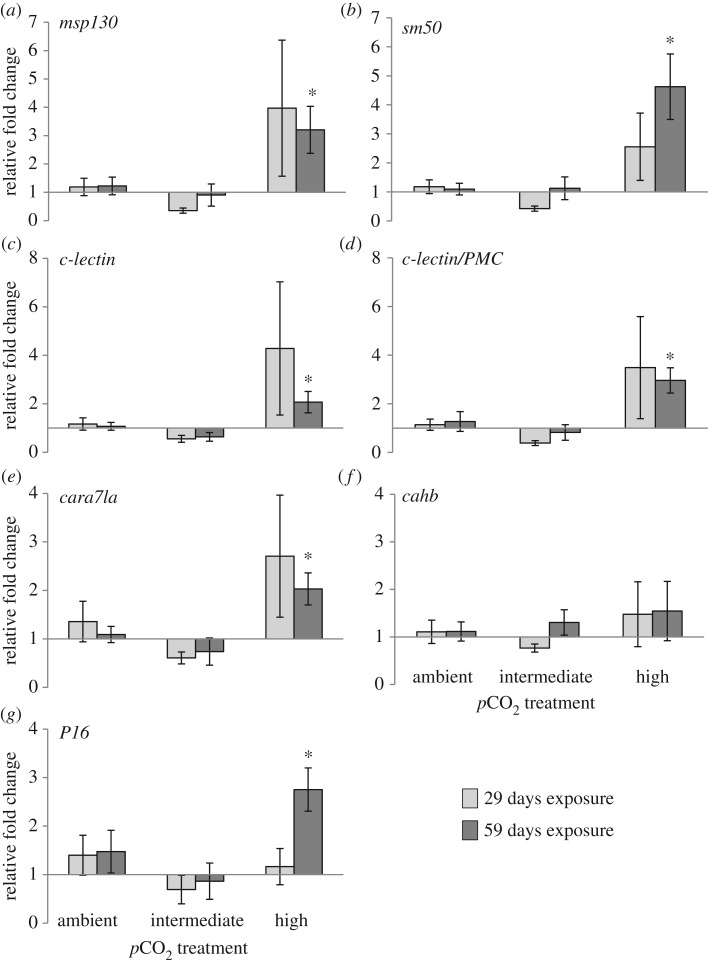
Expression of biomineralization genes in regenerating spines from adult sea urchins exposed to elevated *p*CO_2_ for 29 days (light grey bars) and 59 days (dark grey bars). Data are means ± s.e.m., *n* = 6 in each treatment group, except *n* = 4 for intermediate treatment at 29-day exposure and *n* = 5 for high treatment at 59-day exposure. Relative fold change = (*E*_target_^ΔCt^ ^(mean ambient − sample)^)/(*E*_reference_^ΔCt^ ^(mean ambient − sample)^), geometric mean from three reference genes (*rpl8*, *profilin* and *cyclophilin7*); *significantly higher than ambient (one-way ANOVA or Kruskal–Wallis, *p* < 0.05, *post hoc* multiple range test).

Measurement of the expression of genes involved in biomineralization (*msp130*, *c-lectin*, *c-lectin/pmc1*, *sm50*, *cahb*, *cara7la* and *P16*) in regenerating spines exposed to elevated *p*CO_2_ conditions for 29 days indicated a pattern of upregulation for most of these genes in the high treatment group, but this pattern was non-significant (figure 5, one-way ANOVA, *p* > 0.05). After 59 days exposure to elevated *p*CO_2_ conditions, gene expression in regenerating spines indicated significant upregulation for most of the biomineralization genes (*msp130*, *sm50*, *P16*, *cara7la*, *c-lectin* and *c-lectin/pmc1*) in the high treatment group (figure 5, one-way ANOVA, *p* < 0.05, *post hoc* Fisher's LSD), while carbonic anhydrase b (*cahb*) demonstrated no indication of upregulation (figure 5, *p* > 0.05, one-way ANOVA). Large inter-individual variation existed in the gene expression values within treatment groups. In particular, one individual in the high *p*CO_2_ treatment group did not display upregulation of any of the candidate genes, in contrast with the other individuals in the high *p*CO_2_ treatment group.

Expression of biomineralization genes in regenerating tube feet collected at 29 and 59 days exposure, respectively, was measured using pooled cDNA from the high *p*CO_2_ condition relative to the ambient condition. There were no large differences in gene expression, but statistical significance could not be investigated as samples were pooled (electronic supplementary material, figure S4*a*,*b*). The genes that showed an indication of upregulation in the high *p*CO_2_ group relative to the ambient control at 59 days exposure (*cahb*, *c-lectin*, *c-lectin/pmc1*) were tested using the individual samples. Although there was an increase in expression for all three genes in the high treatment group, the results were not significant (electronic supplementary material, figure S4*c*, one-way ANOVA, *p* > 0.05).

### 3.6. Physiological state

Time taken for sea urchins to ‘right’ to a normal position following inversion was measured as an indicator of physiological fitness at 29 days exposure and 58 days exposure. Righting response time was not affected by *p*CO_2_ treatment (electronic supplementary material, figure S5*a*, one-way ANOVA, *p* > 0.05). Sea urchin weight gain over 58 days of exposure was also unaffected by elevated *p*CO_2_, with no significant difference in initial weight or final weight between *p*CO_2_ treatment groups (electronic supplementary material, figure S5*b*, GLM, *p* > 0.05). Total coelomocyte cell concentration and percentage red cells (electronic supplementary material, figure S5*c*) were not affected by *p*CO_2_ treatment (GLM, *p* > 0.05).

## 4. Discussion

Sea urchins and other echinoderms have a tremendous potential for tissue regeneration. The ability to regenerate damaged spines and tube feet allows sea urchins to maintain critical functions such as locomotion, feeding, sensing the environment and defence against predators, and therefore has a direct impact on survival in the wild. It has been hypothesized that regenerative processes involving biomineralization will be negatively impacted by ocean acidification [8]; however, our study of *L. variegatus* suggests that the rate of regeneration of both spines and tube feet was maintained throughout two rounds of regeneration during the 59 day exposure to low pH. The results are consistent with those observed for the sea star *Luidia clathrata*, which also maintained arm regeneration rates upon exposure to increased *p*CO_2_ [30]. Previous studies using brittlestars exposed to low pH have yielded conflicting results. While one study demonstrated metabolic depression and an 80% reduction in arm regeneration, other studies have shown increased rates of metabolism and either no effect or enhanced arm regeneration under conditions of decreased pH [31–33]. The impact of increased *p*CO_2_ on metabolism in sea urchins was not measured in this study, but it would be important to determine if there is a metabolic cost associated with maintenance of the rate of regeneration under low pH conditions.

Despite the observation that the rate of regeneration was maintained in sea urchin tissues with decreasing pH, the structural integrity and strength of both the regenerating and homeostatic spines appeared negatively affected in the high *p*CO_2_ treatment group. The regenerating spines were not fully regrown at the conclusion of the experiment (70–75% at 59 days of exposure) and therefore it is not surprising that the pores of the stereom are larger and the septa thinner than that of the homeostatic spines. However, the differences in the regenerating spine structure are more pronounced in the high *p*CO_2_ conditions indicating that thickening of the septa and filling in of the pores are highly compromised at low pH. The higher magnesium to calcium ratio observed in regenerating spines is consistent with the observation that the amorphous CaCO_3_ phase of the growing spine favours higher incorporation of magnesium, whereas less magnesium is incorporated in the later stages of spine formation [34]. The higher magnesium content in regenerating spines may result in higher solubility, further contributing to the compromised structure of the regenerating spines. Calcification in regenerating spines takes place within a closed syncytia and therefore the skeleton is not in direct contact with seawater. However, calcification could be impacted indirectly by the cost of maintaining acid–base balance or through dissolution as a result of permeability of the epidermis. The magnesium content of the spines in this study ranged from 5.04 to 5.63 mol%, a value that is lower than the reported average value of 7.2 mol% for spines from *L. variegatus* collected in Florida [35]. However, the magnesium content reported by Magdans & Gies [35] decreased along the length of the spine from the base (7.6 mol%) to the tip (4.9 mol%), and elemental analysis in this study used only 1 cm of the spine measured from the tip, which could explain this discrepancy.

The larger pores and thinner septa of the regenerating spine microstructure resulted in reduced mechanical strength; however, it is interesting that the strength of the homeostatic spines exposed to high *p*CO_2_ conditions was also significantly compromised even though the structural differences were less pronounced. Consistent with our results, previous studies have found increased fragility of the spines of *Tripneustes ventricosus* exposed to high *p*CO_2_ for five weeks, and *Strongylocentrotus droebachiensis* exposed for six weeks, which may be attributed to dissolution as a result of contact with seawater via fractures in the outer epidermis [10,36]. In this study, the electron micrographs show some evidence of dissolution with a reduction in the sharpness of the external barbs on the homeostatic spines of *L. variegatus*.

The sea urchin endoskeleton is made up of a close association between the Mg-calcite structure and a diverse array of extracellular occluded proteins such as SM50, MSP130 and P16 [17–19]. The expression of genes encoding these proteins has been shown to be affected by exposure to elevated *p*CO_2_ in sea urchin larvae of various species, but with no clear consensus of up- or downregulation [37–41]. Gene expression changes in response to ocean acidification have not been investigated in early life stages of *L. variegatus*, but results of this study suggest impacts of elevated *p*CO_2_ on transcription in adult tissues. The expression of several biomineralization-related genes (*msp130*, *sm50*, *P16*, *cara7la*, *c-lectin* and *c-lectin/pmc1*) was significantly upregulated in regenerating spines in the high *p*CO_2_ treatment group relative to the ambient control, indicating compensation at the molecular level for lowered carbonate saturation state. Significant upregulation in expression of biomineralization genes was not seen in sea urchin tube feet; however, this may be because the calcified structure constitutes only a small portion of this tissue. Changes in biomineralization gene expression in tube feet may be evident if mRNA is preferentially extracted from the distal disc that contains the calcium ossicles.

Large inter-individual variation in gene expression was observed within treatment groups. This variability may indicate that a subset of the population is better equipped to cope with ocean acidification, allowing the species to adapt to challenging environmental conditions. Previous studies have shown that offspring of sea urchins (*Paracentrotus lividus*) living in challenging environments with low pH may be more resistant to ocean acidification than those raised in ambient pH conditions [42], and other studies have shown that Antarctic sea urchins (*Sterechinus neumayeri*) can acclimate to reduced pH conditions following long-term exposures, with no negative effects on metabolism, reproductive endpoints or skeletal mass [43]. Sea urchin larvae (*Strongylocentrotus purpuratus*) have the capacity to undergo rapid evolution when facing conditions of ocean acidification [44]. High variation in local carbonate chemistry, high genetic diversity within the population, high fecundity and high dispersal potential have been proposed as likely conditions for genetic adaptation to occur in response to ocean acidification [44]. The high degree of genetic heterogeneity between individual sea urchins may have contributed to the large inter-individual variation observed in the gene expression results in this study. Additionally, the high inter-individual variability may be caused by a mixed population of sea urchins with varying degrees of exposure and adaptation to different CO_2_ conditions. The CO_2_ dynamics of Mangrove Bay, the environment where the sea urchins used in this study were collected, have been well characterized and show large diel variations in *p*CO_2_, ranging from below 500 µatm to approximately 4200 µatm (pH ranges of 7.2–8.1) [45,46]. It is conceivable that long- and short-term residents in our collection location may exhibit varying degrees of adaptation to variable CO_2_ conditions. Although sea urchin movement has not been studied extensively in Bermuda, low recapture rates following calcein tagging for the purpose of measuring growth rate indicated that there is a high degree of migration of *L. variegatus* in Bermuda [47]. Although the sea urchins that reside in Mangrove Bay experience a large diel range in *p*CO_2_, the conditions in this study were measuring their response to sustained exposure to low pH over an extended period of eight weeks.

Righting response, the time taken to ‘right’ to a normal position following inversion, has been used as an indicator of stress and physiological fitness in sea urchins. No significant difference in righting response was found in the increased *p*CO_2_ treatment groups relative to the control group, consistent with previous results showing that righting response was not impacted in juvenile sea urchins (*L. variegatus*) or adult sea stars (*Luidia clathrate*) exposed to elevated *p*CO_2_ [30,48]. Previous studies using juvenile *L. variegatus* found reduced growth rate during three-month exposures to elevated *p*CO_2_; however, the mean weights of animals in high *p*CO_2_ groups did not differ from controls for the first eight weeks of treatment [9] consistent with the results of this study. Growth rate was not affected in adult *Hemicentrotus pulcherrimus* exposed to elevated *p*CO_2_ over nine months; although food intake was reduced and gonadal maturation and spawning were delayed by one month indicating an effect on reproductive processes [11]. Longer exposure times and a wider range of physiological endpoints should be investigated in future studies of *L. variegatus* exposed to reduced pH.

Previous studies have reported both increases and decreases in sea urchin immune cell concentration, and changes in composition of coelomocyte subpopulations in response to elevated *p*CO_2_ [49,50]. In addition, the percentage of red spherule cells, a particular subpopulation of coelomocytes, has been shown to increase in sea urchins in response to stress [51]. In this study, there were no observed effects of increased *p*CO_2_ on coelomocyte concentration or the percentage of red spherule cells. Further research is necessary to fully elucidate the complex response of the immune system to changing environmental conditions.

In summary, sea urchins provide a powerful model organism to understand effects of ocean acidification on the critical role of regeneration and biomineralization in maintaining form and function. Maintenance of the rate of regeneration of spines and tube feet during prolonged exposure to elevated *p*CO_2_ indicates that these processes are a priority for the organism even under stressful conditions. However, compromised spine integrity indicates that they cannot fully compensate for the altered carbonate chemistry. The high inter-individual variation observed in response to elevated *p*CO_2_ may be the result of natural genetic diversity and suggests that a subset of the population is better able to adapt to environmental stress. Future studies should focus on understanding the heterogeneous response to CO_2_-driven ocean acidification by studying a larger number of individuals and using genetically isolated populations of sea urchins. Full transcriptome or proteome profiling could lead to a more complete understanding of the molecular responses and capacity for compensatory mechanisms to alleviate effects of increased *p*CO_2_. In addition, results should be interpreted within the context of the naturally high and variable *p*CO_2_ conditions that are experienced in coastal ecosystems [45,46]. Ultimately, it will be vital to determine the ecological impact of increased spine fragility and compromised integrity of regenerating spines on essential functions such as protection against predation, as well as to determine the biological consequences of increased allocation of resources to calcification. Understanding how sea urchins respond to stressful environmental conditions, such as those presented by CO_2_-driven ocean acidification, will provide insight into their resiliency and enable predictions of future outcomes of environmental change on populations.

## Supplementary Material

Spine SNAP Test

## Supplementary Material

Seawater pH

## Supplementary Material

Seawater parameters

## Supplementary Material

L. variegatus tube feet gene expression

## Supplementary Material

Physiological state of sea urchins

## Supplementary Material

Lv QPCR primer sequences

## Data accessibility

The data supporting this article have been uploaded as part of the supplementary material and are available from the Dryad Digital Repository (http://dx.doi.org/10.5061/dryad.f6r10) [52].
