# Supplementary material for: Ocean acidification impacts spine integrity but not regenerative capacity of spines and tube feet in adult sea urchins
Source: R Soc Open Sci. 2017 May 17;4(5):170140. doi: 10.1098/rsos.170140 (PMC5451823; doi:10.1098/rsos.170140)
Supplement: Spine SNAP Test [file rsos170140supp1.docx]

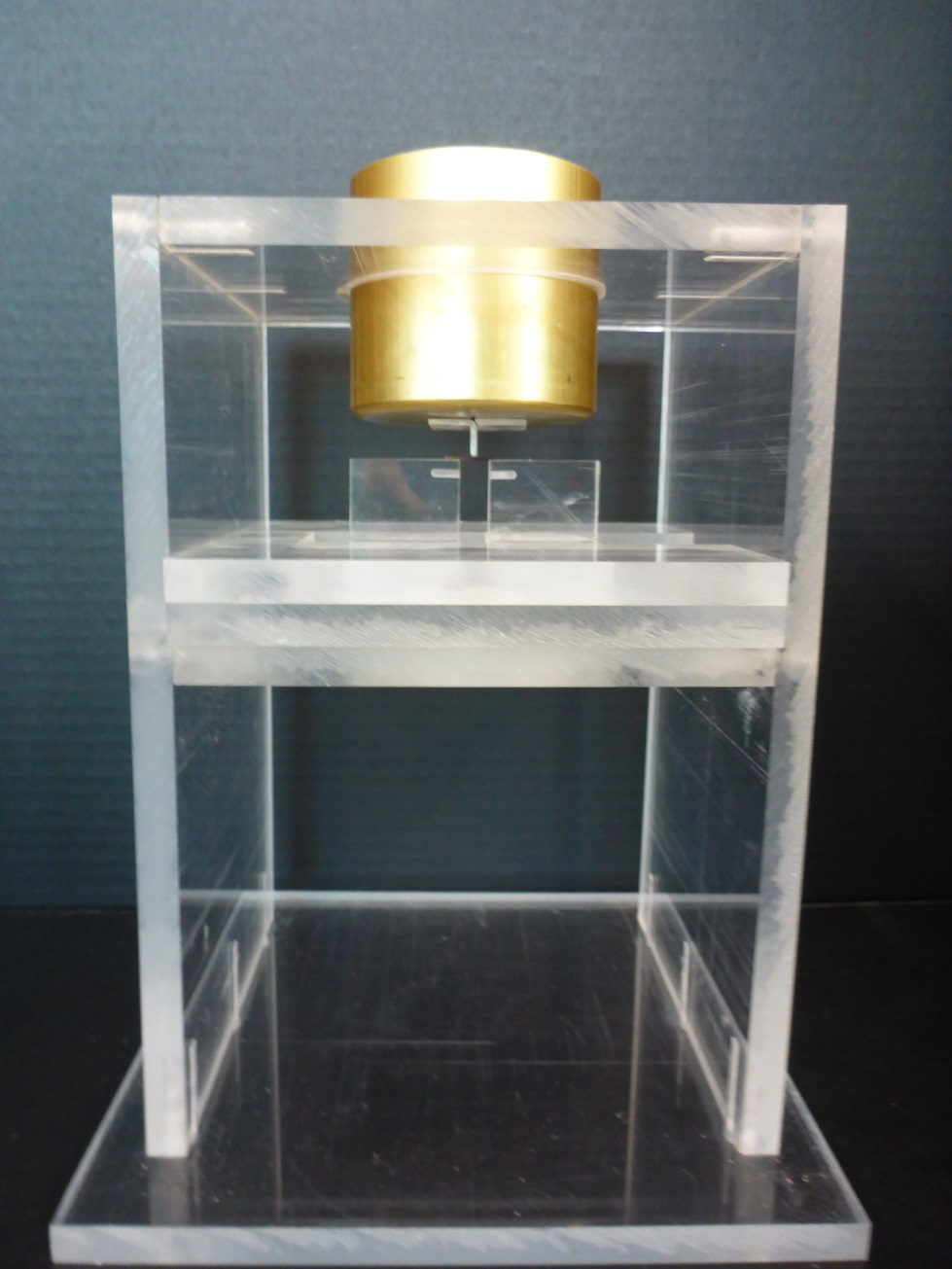

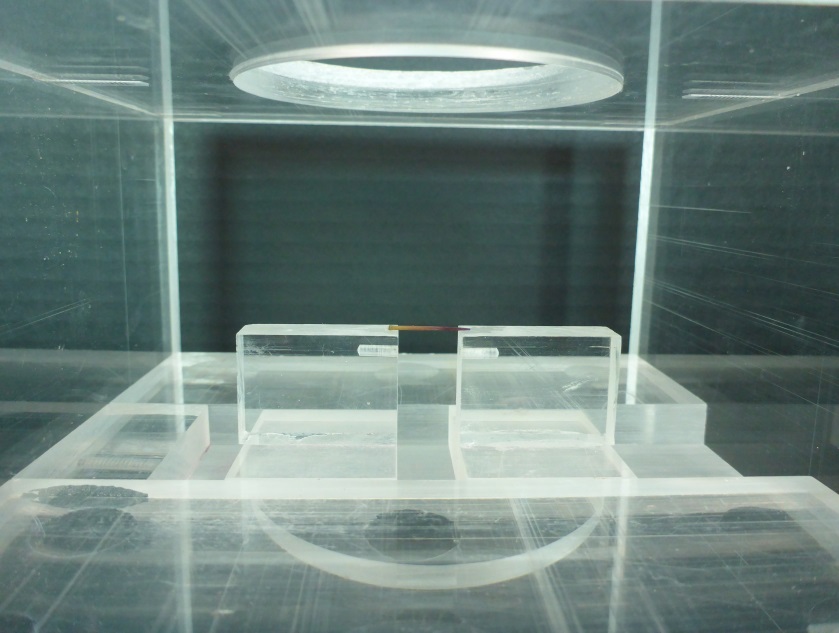


**S**

**R**

**++++**

**Figure S1.** Mechanical loading assay for sea urchin spine snap test. Spine (S) of standardized length (9 mm) is balanced between two platforms so that 1 mm rests on each platform and 7 mm was left unsupported. The receptacle (R) is placed in the middle of the spine and small lead pellets (0.1-0.2 grams each) are added into the receptacle until the spine snaps. The receptacle and load are weighed to determine the load bearing weight. The inset on the bottom right shows an enlarged image of a spine balanced between the two platforms.
