# Supplementary material for: Ocean acidification impacts spine integrity but not regenerative capacity of spines and tube feet in adult sea urchins
Source: R Soc Open Sci. 2017 May 17;4(5):170140. doi: 10.1098/rsos.170140 (PMC5451823; doi:10.1098/rsos.170140)
Supplement: Seawater pH [file rsos170140supp2.docx]

**Figure S2.** Seawater pH. Seawater from each treatment tank (ambient treatment is black diamonds, intermediate *p*CO_2_ treatment is dark grey diamonds, high *p*CO_2_ treatment is light grey diamonds) was sampled weekly over the 62 day experiment. Water was analyzed for dissolved inorganic carbon and total alkalinity from which pH was calculated.
