# Supplementary material for: Ocean acidification impacts spine integrity but not regenerative capacity of spines and tube feet in adult sea urchins
Source: R Soc Open Sci. 2017 May 17;4(5):170140. doi: 10.1098/rsos.170140 (PMC5451823; doi:10.1098/rsos.170140)
Supplement: Seawater parameters [file rsos170140supp3.docx]

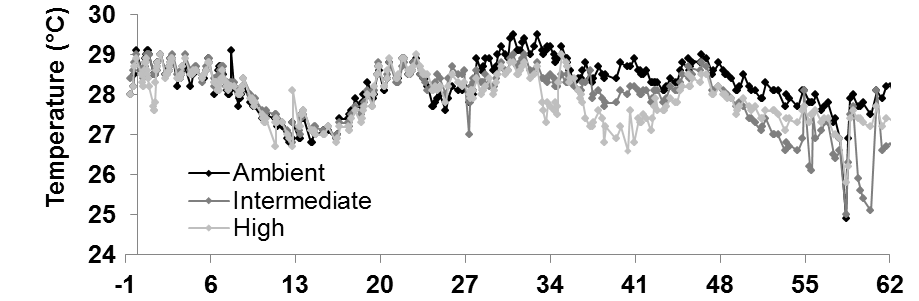


**Exposure time (days)**


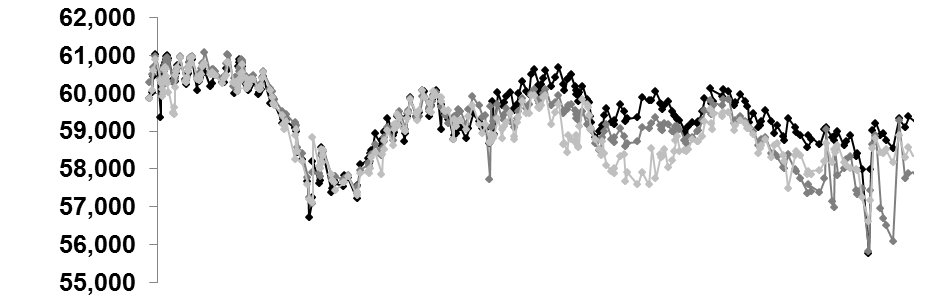

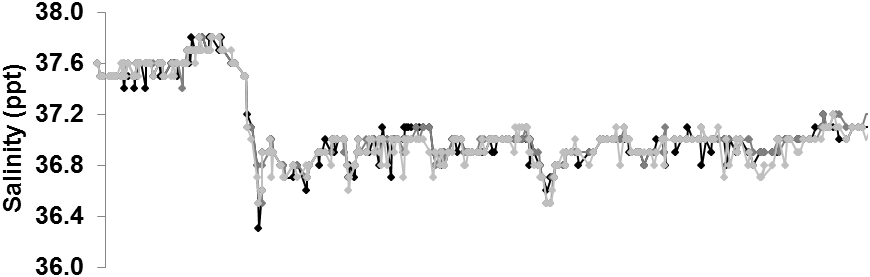


**a**

**b**

**c**

**Conductivity (µS/cm)**

**Figure S3.** Experimental seawater conditions. Seawater parameters including conductivity (a), salinity (b), and temperature (c) were measured multiple times each day over the 62 day experiment in each treatment tank (ambient treatment is black diamonds, intermediate *p*CO_2_ treatment is dark grey diamonds, high *p*CO_2_ treatment is light grey diamonds). Vertical dashed lines indicate initial spine and tube feet amputation (day 0), re-amputation (day 30 exposure), and final regeneration measurement (day 59 exposure).
