# Supplementary material for: Ocean acidification impacts spine integrity but not regenerative capacity of spines and tube feet in adult sea urchins
Source: R Soc Open Sci. 2017 May 17;4(5):170140. doi: 10.1098/rsos.170140 (PMC5451823; doi:10.1098/rsos.170140)
Supplement: L. variegatus tube feet gene expression [file rsos170140supp4.docx]

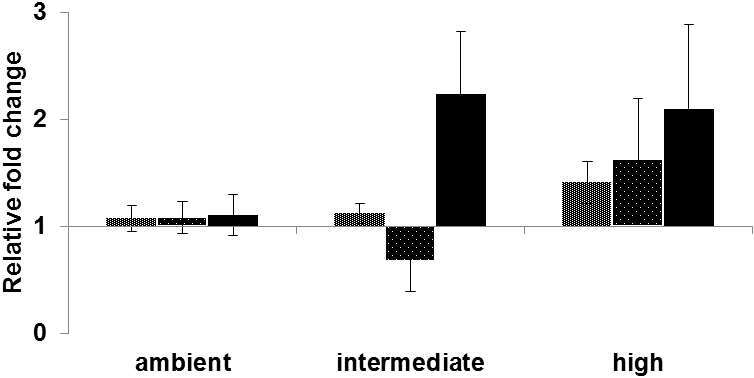

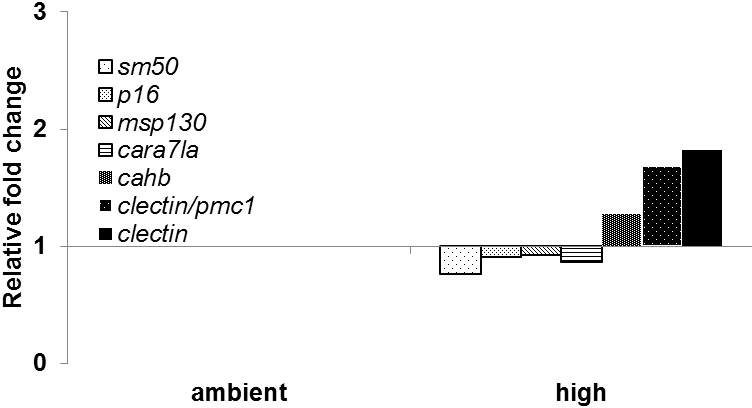


**a –** 29 d exposure, pooled samples

**b –** 59 d exposure, pooled samples

**c –** 59 d exposure, all samples


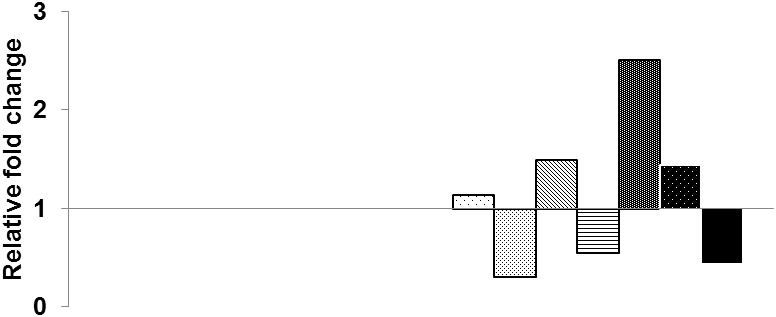


**ambient high**

**Figure S4.** Tube feet gene expression. Expression of biomineralization genes in regenerating tube feet from sea urchins exposed to elevated *p*CO_2_ for 29 days (a) and 59 days (b and c). Panels a and b show results from pooled samples (n = 6 individuals per treatment); panel c represents means ± s.e.m., n=6 individuals per treatment. Relative fold change = (*E*_target_^ ^Ct^ ^(mean ambient -sample)^) / (*E*_reference_^ ^Ct^ ^(mean ambient - sample)^), geometric mean from three reference genes (*rpl8*, *profilin*, and *cyclophilin7*). No significant differences were observed (one-way ANOVA, p > 0.05).
