# Supplementary material for: Ocean acidification impacts spine integrity but not regenerative capacity of spines and tube feet in adult sea urchins
Source: R Soc Open Sci. 2017 May 17;4(5):170140. doi: 10.1098/rsos.170140 (PMC5451823; doi:10.1098/rsos.170140)
Supplement: Physiological state of sea urchins [file rsos170140supp5.docx]

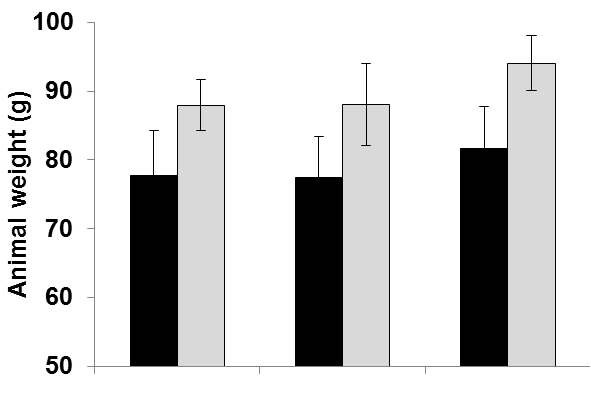


**a**


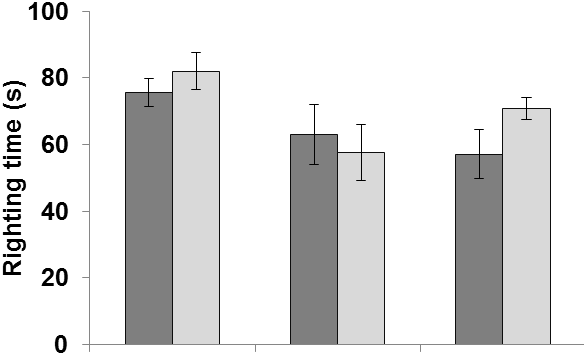


**b**


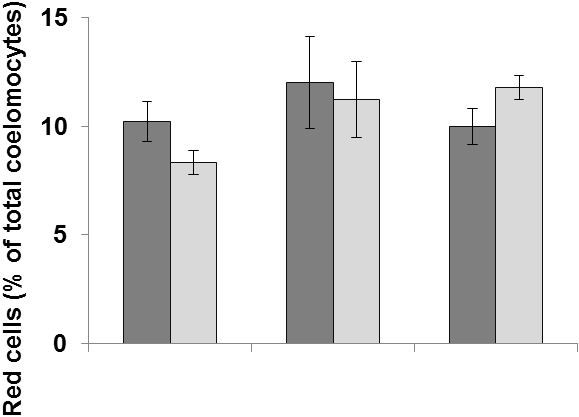


**Ambient Intermediate High**

***p*CO_2_ treatment**

**c**

0 day exposure

28-29 day exposure

58 day exposure

**Figure S5.** Physiological effects of *p*CO_2_ treatment on sea urchins. Righting time (a), growth (b), and proportion of red coelomocytes (c) were all unaffected by *p*CO_2_ treatment, GLM, p > 0.05. Data are means ± s.e.m., n=6 animals per treatment (n=5, righting time intermediate treatment, p<0.05, Grubb’s outlier test).
