# Supplementary material for: Ocean acidification impacts spine integrity but not regenerative capacity of spines and tube feet in adult sea urchins
Source: R Soc Open Sci. 2017 May 17;4(5):170140. doi: 10.1098/rsos.170140 (PMC5451823; doi:10.1098/rsos.170140)
Supplement: Lv QPCR primer sequences [file rsos170140supp6.docx]

**Table S1.** qRT-PCR primer details for biomineralization and control genes.

| **Gene name** | **Gene identifier^a^** | **Forward primer (5’-3’)** | **Reverse primer (5’-3’)** |
| --- | --- | --- | --- |
| **Biomineralization genes** | | |  |
| *c-lectin/pmc1* | LVA_014662 | GAAGGTGCTAGTGGCGATCCT | TGTCTGAGGGCCGTTGCT |
| *c-lectin* | LVA_021331 | AGCTGGCGAACCCAACAA | TCAACACTCATCTGCGCGTAGT |
| *cahb* | LVA_0114119 | CCCGTCCCGAATCTTCAAG | CCAGGGTTGCGAATGATGA |
| *cara7la* | SPU_012518 | CCACGGACAACCATGTCAATAA | TCTACTTTGGTCGTAGGAGCTCTGT |
| *P16* | SPU_018408 | GCAGCGATGACTCGTCAGAAG | GCCAAAGGCCATACCACTCTT |
| *sm50* | SPU_018811 | GTACCATCTTCCCAGAATCTAGGATT | AAGCTTCTCGCAAGACAACCA |
| *msp130* | LVA_019583 | CTACCACACCGCTACCAGGAA | TCTGGGTGACTACGTAGCTCATG |
| **Control genes** | | | |
| *cyclophilin-7* | SPU_008305 | CCTCCTTCCACAGGGTTATCC | GTACCGTTGCCCCTGGTAAA |
| *rpl8* | SPU_010692 | GCCAACAGGGCCATGGT | TTACGCTTGACCTTGTATTTGAAGTAG |
| *profilin* | SPU_020197 | TGCAGGCGAGTAAGACAGCTATA | CTCCTTTATTCAAGTTCCCTTGCT |

**^a^** Gene identifiers from annotated genome of *S. purpuratus* and *L. variegatus* ([www.echinobase.org](http://www.echinobase.org)).
